# Stability of spectral estimates in resting-state magnetoencephalography: Recommendations for minimal data duration with neuroanatomical specificity

**DOI:** 10.1016/j.neuroimage.2021.118823

**Published:** 2021-12-16

**Authors:** Alex I. Wiesman, Jason da Silva Castanheira, Sylvain Baillet

**Affiliations:** McConnell Brain Imaging Centre, Montreal Neurological Institute, McGill University, Montreal QC, Canada

**Keywords:** Neural oscillations, Magnetoencephalography, Signal variability, Spectral analyses, Robust estimation

## Abstract

The principle of resting-state paradigms is appealing and practical for collecting data from impaired patients and special populations, especially if data collection times can be minimized. To achieve this goal, researchers need to ensure estimated signal features of interest are robust. In electro- and magnetoencephalography (EEG, MEG) we are not aware of any studies of the minimal length of data required to yield a robust one-session snapshot of the frequency-spectrum derivatives that are typically used to characterize the complex dynamics of the brain’s resting-state. We aimed to fill this knowledge gap by studying the stability of common spectra measures of resting-state MEG source time series obtained from large samples of single-session recordings from shared data repositories featuring different recording conditions and instrument technologies (OMEGA: *N* = 107; Cam-CAN: *N* = 50). We discovered that the rhythmic and arrhythmic spectral properties of intrinsic brain activity can be robustly estimated in most cortical regions when derived from relatively short segments of 30-s to 120-s of resting-state data, regardless of instrument technology and resting-state paradigm. Using an adapted leave-one-out approach and Bayesian analysis, we also provide evidence that the stability of spectral features over time is unaffected by age, sex, handedness, and general cognitive function. In summary, short MEG sessions are sufficient to yield robust estimates of frequency-defined brain activity during resting-state. This study may help guide future empirical designs in the field, particularly when recording times need to be minimized, such as with patient or special populations.

## 1. Introduction

The study of intrinsic brain activity during resting-state is advancing our understanding of neural processes underlying a wide spectrum of brain functions in health and disease. Though the full functional relevance of the approach is still debated (Cole et al., 2010; Gonzalez-Castillo et al., 2021; Stevens and Spreng, 2014), the potential benefits of task-free paradigms are multifold. In electro- and magnetoencephalography (EEG, MEG), resting-state protocols lend themselves to a great variety of analysis approaches based on spectral transformations, including sophisticated derivatives of cross-frequency interactions (Canolty and Knight, 2010; Florin and Baillet, 2015) and functional connectivity (Hutchison et al., 2013). In principle, the fast temporal resolution of EEG and MEG may enable the estimation of such metrics across time, possibly over short, sliding time windows. A companion idea is to determine core individual features of brain activity in single individuals from the minimum possible amount of data, i.e., recording duration, especially in paediatric populations (Kurz et al., 2014, 2018; Raschle et al., 2012), those with chronic pain (Davis et al., 2017; Diers et al., 2020; Witjes et al., 2021), and patients with cognitive impairment (Binnewijzend et al., 2012; Cassani et al., 2018; Mandal et al., 2018; Rombouts et al., 2005; Wiesman et al., 2021a, 2021b, 2018). Both aspects require the rigorous determination of brain signal stationarity and robustness of feature extraction.

Intra-session variability in resting-state activity has been of recent interest for “fingerprinting” (da Silva Castanheira et al., 2021; Finn et al., 2015), functional parcellation (Laumann et al., 2015), and enhanced prediction of behaviour (Waschke et al., 2021) at the level of individual participants. Notwithstanding these recent efforts, most resting-state research derives and interprets summary statistics of neural fluctuations over data durations that vary considerably between studies, ranging at least from one (Ranasinghe et al., 2020) to fifteen (Marquetand et al., 2019) minutes. We are not aware of systematic studies of the within-session robustness of commonly used estimates of resting-state MEG signal features, nor of any resulting guidelines for minimum recording durations. Such guidelines would contribute to the evidence-based design of resting-state MEG studies that are more likely to be reproducible and would help researchers make informed decisions concerning key data collection parameters of shared repositories of MEG resting-state data.

Previous electrophysiological reliability studies have focused on the consistency of various signal metrics across sessions separated by days, months, or years (Duan et al., 2021; Fingelkurts et al., 2006; Garcés et al., 2016; Gasser et al., 1985; Keil et al., 2003; Lew et al., 2021; Liuzzi et al., 2017; Marquetand et al., 2019; McCusker et al., 2021; Thatcher, 2010). Here, we instead investigate the minimum recording duration required to achieve robust estimates of the main spectral properties of MEG resting-state source signals *within a single recording session*. Thus, we define a robust estimate as a signal derivative that exhibits stable, consistent values when computed from a sufficiently long duration of data recorded during a single session.

We also emphasize the recent interest in discriminating between periodic (i.e., rhythmic) and aperiodic (i.e., arrhythmic) components of the power spectrum of neurophysiological brain signals (Donoghue et al., 2020). Studies have shown that their respective parameterizations help clarify the possible confounding contribution of background arhythmic brain activity to the measurement of oscillatory components of neural activity (Donoghue et al., 2021). For example, variability in the aperiodic slope and offset accounts for changes in the neural power spectrum along the lifespan (Cellier et al., 2021; Schaworonkow and Voytek, 2021), covaries with clinical outcomes in a number of disorders (Ostlund et al., 2021; Pani et al., 2021; Van Heumen et al., 2021; Wilkinson and Nelson, 2021), and predicts memory consolidation during sleep (Helfrich et al., 2021) and individual differences in visuomotor learning (Immink et al., 2021). Regarding the reliability and stability of these components, Pathania et al. (2021) found that estimates of aperiodic components are consistent over intervals of at least seven days. We wish to expand these investigations by determining the minimum data duration required to produce robust estimates of the periodic and aperiodic components of MEG resting-state source maps.

Here we qualify an estimate obtained from a given data length as robust when its group-wise consistency exceeds pre-defined thresholds across measurements. We use intra-class coefficient (ICC) statistics, with thresholds of ICC > 0.90 for excellent, ICC > 0.75 for good, and ICC > 0.50 for moderate levels of estimate stability (Koo and Li, 2016). We examine the stability of spectral features derived from varying lengths of source-imaged MEG resting-state data collected from two different models of MEG instrument and available from two different shared repositories: the Open MEG Archive (OMEGA; CTF instrument; *N* = 107; Nisoet al., 2016) and the Cambridge Centre for Ageing and Neuroscience dataset (Cam-CAN; MEGIN/Elekta instrument; *N* = 50; Taylor et al., 2017). We use the Fitting Oscillations & One-Over-F toolbox (FOOOF; Donoghue et al., 2020) to decompose the spectra from MEG source time series into periodic and aperiodic components and examine the robustness of these components over varying data durations. Finally, we leverage the balanced demographic distribution and detailed cognitive testing of the Cam-CAN dataset to investigate potential linear effects of key demographics (i.e., age, sex, and handedness) and cognitive function (as measured by the Addenbrooke’s Cognitive Examination – Revised; ACE-R; Mioshi et al., 2006) on the intra-session stability of MEG resting-state spectral features, derived from multiple tested data durations. From these empirical results, we recommend minimum data durations, contingent on spectral and neuroanatomical features of interest, to ensure the robustness of spectral estimates from MEG resting-state data.

## 2. Methods

### 2.1. Data and participants

We used subsets of data from two shared repositories: the Open MEG Archive (OMEGA; Nisoet al., 2016) and the Cambridge Centre for Ageing and Neuroscience dataset (Cam-CAN; Shafto et al., 2014; Taylor et al., 2017). Both OMEGA and Cam-CAN include resting-state MEG recordings from healthy adults, alongside basic demographic data and T1 MRIs. Key differences in the resting-state acquisition parameters between the two datasets include: the MEG system used (OMEGA: 275-axial gradiometer CTF, Port Coquitlam, BC, Canada; Cam-CAN: 204-planar gradiometer & 102-magnetometer MEGIN/Elekta VectorView, Helsinki, Finland), the data acquisition site (OMEGA: Montreal, QC, CA; Cam-CAN: Cambridge, UK), basic participant behaviour (OMEGA: eyes-open; Cam-CAN: eyes-closed), and the sampling rate (OMEGA: 2400 Hz; Cam-CAN: 1000 Hz).

From the OMEGA dataset, 107 participants (mean age = 30.57 [SD = 12.70]; age range = 19 – 74 years; 102 right-handed; 46 female) were included based on the following criteria: no current neurological or psychiatric disorder, no history of head trauma, and no MRI or MEG contraindications. Participants without MEG, MRI, or demographic data (or any auxiliary data necessary for the present analyses; e.g., head digitization files for MEG) were also excluded. Eyes-open resting-state MEG data were collected from each participant at a sampling rate of 2400 Hz and with an antialiasing filter with a 600 Hz cut-off. Noise-cancellation was applied using CTF’s software-based built-in third-order spatial gradient noise filters. Recordings lasted a minimum of 4 min and were conducted with participants in the seated position as they fixated on a centrally-presented crosshair.

**Fig. 1.**
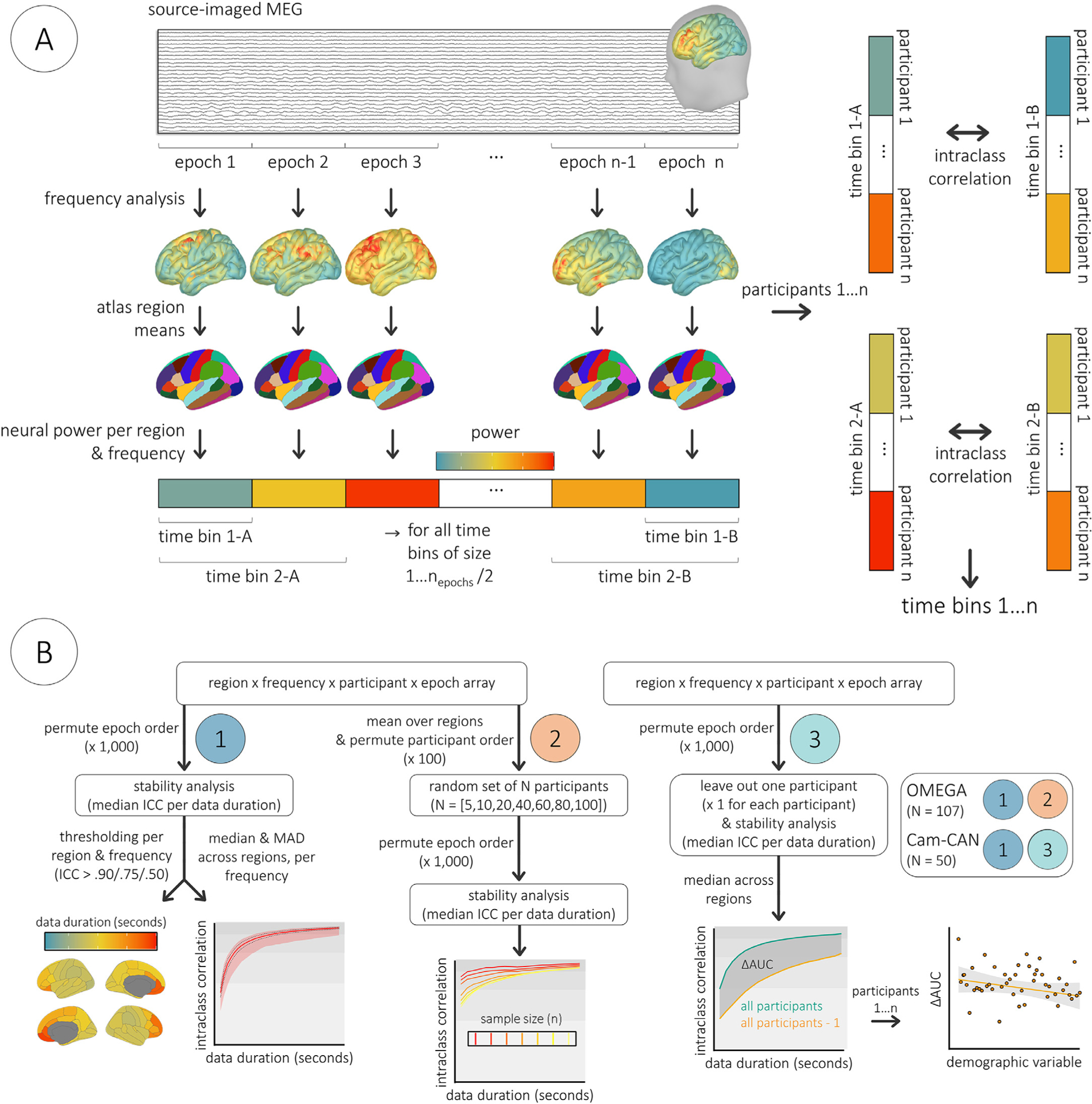
Stability analysis pipeline. Source-imaged MEG data was epoched into consecutive, non-overlapping segments and transformed into the frequency domain per vertex. Power spectral density values were then averaged within canonical frequency bands and over vertices within Desikan-Killiany atlas regions. For each of 1000 permutations, the temporal order of these epochs was randomized. Data for each cortical location, frequency, and participant were then averaged over time bins of progressively larger numbers of epochs from one to n_epochs_/2 and used to compute ICC with time bins averaged over the same number of different epochs. For instance, within each permutation for participants 1…n, spectral power estimates from the first epoch (time bin 1-A) were correlated with spectral power estimates derived from the last epoch (time bin 1-B), spectral power estimates averaged over the first two epochs (time bin 2-A) were then correlated with spectral power estimates averaged over the last two epochs (time bin 2-B), and so forth until all epochs were included (two time bins each averaged over n_epochs_/2 epochs, i.e., 1/2 of the total recording duration). Computing the median across these permutations for each size of time bin (from 1 to n_epochs_/2) resulted in a time course of intra-session ICC values per each combination of region and frequency. (B) Graphical workflows. All analysis steps are indicated for the derivation of (1) stability estimates per each combination of region and frequency, (2) stability estimates per each combination of frequency and sample size, and (3) change scores representing the omission of each participant from the stability analysis. The extra inset to the far right indicates the participant subsamples on which each workflow was implemented. AUC: area under the curve. Cam-CAN: Cambridge Centre for Ageing and Neuroscience participant subsample. ICC: intraclass correlation coefficient. MAD: median absolute deviation. OMEGA: Open MEG Archives participant subsample.

**Fig. 2.**
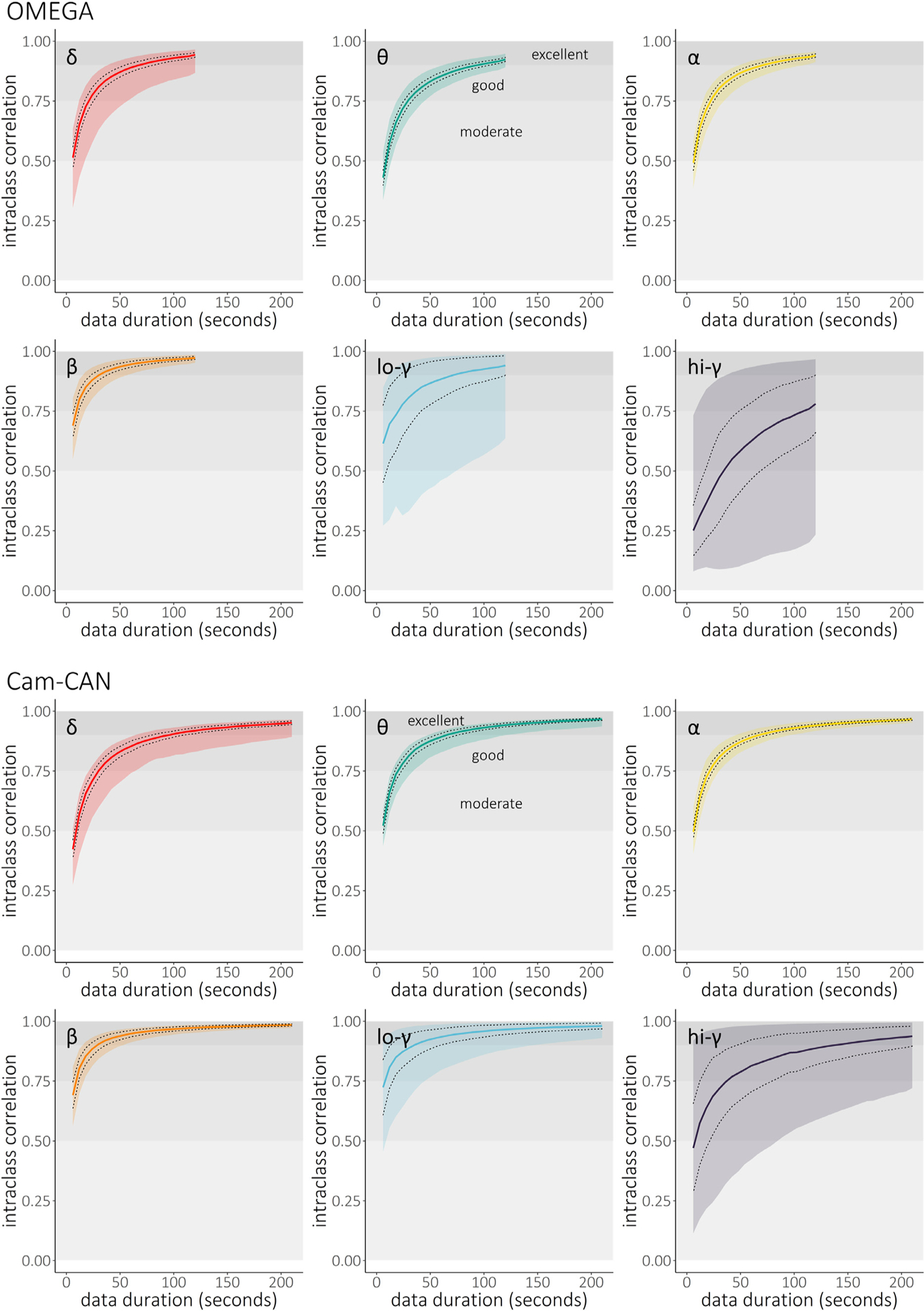
Intra-session temporal stability of band-limited power. Intraclass correlation coefficients (y-axes) were obtained from 1000 permutations of epoch order and represent a measure of the stability of spectral power for each frequency band of interest as a function of data duration (x-axes, in seconds; top: OMEGA, *N* = 107; bottom: Cam-CAN, *N* = 50). Coloured lines represent the median across regions, dotted lines indicate ± one median absolute deviation across regions, and coloured shaded intervals represent the range of stability values across all modelled cortical regions of the Desikan-Killiany atlas. Horizontal shaded intervals in each plot represent typical thresholds used to define moderate (ICC > 0.50), good (ICC > 0.75), and excellent (ICC > 0.90) reliability. Note that the maximum data duration from OMEGA was shorter than from Cam-CAN (see Methods).

We also selected a validation sample of 50 healthy adult participants (mean age = 44.72 [SD = 14.56]; age range = 20 – 69 years; 45 right-handed; 23 female) from the Cam-CAN dataset. The sample size of 50 was determined quasi-empirically, by modelling the time-by-ICC stability relationship as a function of sample size in the OMEGA sample (see 2.4 Temporal stability analysis and Fig. 1 B–2). From these relationships, it was apparent that a sample of at least 40–50 participants was necessary for the time-by-ICC estimation to stabilize, and for the variability in this estimation due to random participant subsampling to diminish (Fig. S1). To study the impact of age on the stability of MEG derivatives, 10 participants were selected per each decade from 20–69 years of age, with the other demographics matched to those of the OMEGA participant sample. Exclusionary criteria included current neurological or psychiatric disorder, MRI or MEG contraindications, unusable MEG, MRI, or demographic data, and cognitive impairment (MMSE ≤ 24). Resting-state MEG data were collected from each participant at a sampling rate of 1000 Hz and with a band-pass filter of 0.03 – 330 Hz. Noise-cancellation was applied using tSSS/MaxFilter (v2.2; 0.98 correlation limit; 10 s window; MEGIN/Elekta). Recordings lasted approximately 8 min with participants in the seated position and their eyes closed.

The data collection and management protocols for the OMEGA and Cam-CAN repositories were approved by the research ethics boards at the Montreal Neurological Institute and the University of Cambridge, respectively. All participants in both studies provided written informed consent in accordance with the Declaration of Helsinki.

### 2.2. MEG data preprocessing

MEG preprocessing procedures for both datasets were similar to those reported by da Silva Castanheira et al. (2021), following good-practice guidelines (Gross et al., 2013). All processing steps were performed using Brainstorm (Tadel et al., 2011), unless otherwise specified. Notch filters were applied at the respective line-in frequency (and harmonics) of each dataset (60 Hz for OMEGA, 50 Hz for Cam-CAN), along with a 0.3 Hz high-pass FIR filter to attenuate slow-wave drift and DC offset. Additional notch filters were applied at 88 Hz (and harmonics) to attenuate known artifacts in the Cam-CAN dataset. Signal space projectors (SSPs) were derived around cardiac and eye-blink events detected from ECG and EOG channels using the automated procedure available in Brainstorm (Niso et al., 2019) and applied to the data. SSPs were also used to attenuate low-frequency (1 – 7 Hz) and high-frequency (40 – 400 Hz) noise related to saccades and muscle activity. To test whether the initial length of the recording influenced the efficacy of these preprocessing steps, and thereby the stability of the underlying spectral derivatives, we replicated all preprocessing (except for tSSS/MaxFilter) and source imaging (see 2.3 Source imaging & spectral analysis) steps on truncated versions of the recordings from the Cam-CAN sample, ranging from 60 to 150 s in increments of 30 s. We then computed our spectral and stability analyses (see 2.3 Source imaging & spectral analysis and 2.4 Temporal stability analysis) with the first 8 six-second epochs extracted from these truncated data segments, limited by the number of available epochs in the 60 s data segment to enhance comparability. To enable juxtaposition with our other analyses, we similarly extracted the 8 corresponding epochs from our “full-length” version of data recordings for stability estimation.

### 2.3. Source imaging and spectral analysis

Using approximately 100 digitized head points, MEG data were co-registered to each participant’s T1-weighted MRI that was segmented and labelled with Freesurfer (Fischl, 2012) using *recon-all*. Source imaging was performed on the full-bandwidth data using only gradiometer data with individually fitted overlapping-spheres forward models (15,000 vertices, with elementary sources constrained normal to the cortical surface) and two widely-used source estimation techniques with Brainstorm default parameters: the linearly-constrained minimum variance (LCMV) beamformer and dynamic statistical parametric mapping (dSPM). dSPM source imaging was performed using noise covariance estimated from empty-room recordings taken as close in time as possible to each participant’s visit. To verify that the inclusion of additional data in the form of empty-room recordings did not bias the stability analysis of dSPM, we also ran a control analysis using the identity matrix in place of the noise covariance matrix. These source imaging procedures yielded continuous time series per each spatially-resolved location (i.e., vertex) on the cortical surface for each participant. Only gradiometer data were used for source imaging to facilitate comparison across the OMEGA and Cam-CAN samples, as the MEG instrument used in the OMEGA study comprises gradiometer sensors only.

Source-imaged data were segmented into contiguous, non-overlapping 6 s epochs, resulting in a total of 40 epochs (total of 240 s) for OMEGA participants and 70 epochs (total of 420 s) for Cam-CAN participants. Vertex-wise estimates of power spectral density (PSD) were obtained using Welch’s method (3 s time window, 50% overlap) and averaged over canonical frequency bands using Brainstorm defaults (delta: 2–4 Hz; theta: 5–7 Hz; alpha: 8–12 Hz; beta: 15–29 Hz; low-gamma: 30–59 Hz; high-gamma: 60–90 Hz; Niso et al., 2019). To examine the potential effect of frequency bandwidth on subsequent analyses, a complementary procedure was performed in the OMEGA participants across the same approximate frequency ranges using constant spectral increments (i.e., from 3–92 Hz in 10-Hz steps). To study the effects of epoch/window length on spectral parametrization (see 2.5 Stability of power spectrum parameterization), we also estimated PSDs from 12 s epochs using 6 s time windows in the OMEGA sample (also with 50% overlap). Within each frequency band and for each participant, PSD values were averaged over vertices belonging to each of the 68 regions of a standard atlas (Desikan et al., 2006) registered to individual structural data. This resulted in an array of PSD values of number of atlas regions × number of frequency bins × number of participants × number of epochs (e.g., 68 × 6 × 107 × 40 from OMEGA).

### 2.4. Temporal stability analysis

The stability of spectral estimates was assessed via intraclass correlation coefficients (ICC; single-rater, two-way mixed-effects, absolute agreement; Koo and Li, 2016; McGraw and Wong, 1996) between multi-dimensional PSD arrays averaged across various numbers of randomly selected, non-overlapping epochs (Fig. 1A). To control for the potential bias that could be due to the epoch’s temporal position in the session, ICC derivations were repeated across 1000 randomizations of the epoch order: for each of 1000 permutations, the order of the matrix data was randomized over epochs using the *randperm* function in MATLAB (version 2019b; Mathworks, Inc., Massachusetts, USA), and ICCs were calculated for each region and frequency between sets of PSD estimates averaged over progressively larger numbers of epochs from one to n_epochs_/2 (using the *ICC.m* function in Matlab; Salarian, 2016). Confidence intervals (95%) were also generated alongside the ICCs to quantitatively compare stability metrics across datasets and analyses. The medians of these values were then computed across randomizations, leading to estimates of intraclass similarity for each cortical region and spectral frequency as a function of recording length (Fig. 1B–1). From these data, the minimum durations where ICC exceeded established thresholds (Koo and Li, 2016) for moderate (ICC > 0.50), good (ICC > 0.75), and excellent (ICC > 0.90) reliability were extracted for each combination of region with frequency and plotted on a spatial representation of the Desikan-Killiany atlas using *ggseg* (Mowinckel and Vidal-Piñeiro, 2020). Additionally, the median, median absolute deviation, and range of ICC values across regions were computed and plotted as a function of time and frequency using *ggplot2* (Wickham, 2011). To examine the potential effect of group size on stability, the original 4-dimensional PSD arrays were averaged across all regions and a similar approach was taken to test for the robustness of spectral estimates across various sample sizes of randomly selected participants (*N* = [5, 10, 20, 40, 60, 80, 100]; Fig. 1B–2). One hundred iterations of the participant randomization were performed and within each of these randomizations the previously described stability estimation approach was followed (1000 epoch permutations). Medians and median absolute deviations of the resulting time-by-ICC estimates were calculated across the 100 participant randomizations to examine their stabilization with increasing sample sizes.

### 2.5. Stability of power spectrum parameterization

We parameterized the power spectra of each brain region using the FOOOF algorithm (Donoghue et al., 2020) implemented in Brainstorm (MATLAB version; frequency range = 0.5–40 Hz; Gaussian peak model; peak width limits = 0.5 – 12 Hz; maximum n_peaks_ = 3; minimum peak height = 3 dB; proximity threshold = 2 SD; fixed aperiodic; no guess weight). Note that gamma-frequencies were not subjected to FOOOF parametrization, as the FOOOF algorithm struggles with fitting PSD data properly above 40 Hz due to the loss of linearity in log-log space and lack of clear peaks in gamma frequency ranges and above (Donoghue et al., 2020). Thus, we elected to use the FOOOF-recommended default range of 0.5–40 Hz. The robustness of estimated aperiodic features from the FOOOF models (i.e., the exponent and offset) over varying epoch lengths was tested using the same procedure detailed above. In addition, the periodic (i.e., rhythmic) features were averaged over the same canonical frequency bands described earlier (see 2.3 Source imaging & spectral analysis) and the robustness over varying epoch lengths tested using the above-mentioned pipeline. To examine whether the goodness of fit of FOOOF models might bias the stability of parameterized features, we regressed each feature on the model fit (i.e., R^2)^ per each relevant location, frequency, and epoch (i.e., with participants as the unit of measurement; using the *fitglm* function in MATLAB) and extracted residuals from these linear models. We then re-ran all FOOOF temporal stability analyses on the fit-corrected FOOOF feature residuals.

### 2.6. Individual stability contribution analysis

**Fig. 3.**
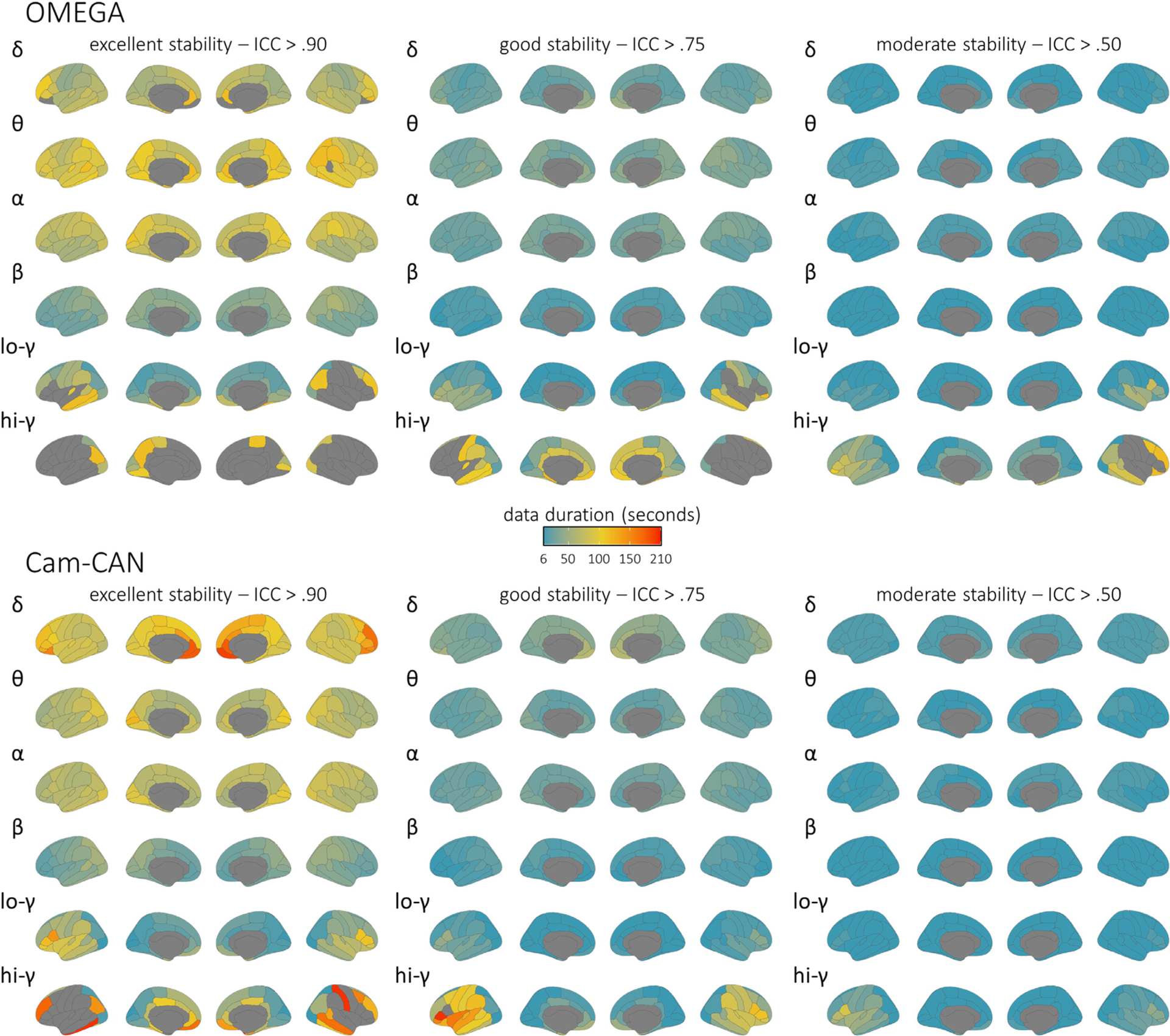
Brain maps of intra-session stability of spectral power estimates. Parcellated surface maps per each spectral frequency (denoted by Greek letters to the top-left of each set) indicate the length of data (colour bar; in seconds) required to achieve accepted thresholds for moderate (ICC > 0.50; right), good (ICC > 0.75; middle), and excellent (ICC > 0.90; left) reliability in each participant sample (top: OMEGA, *N* = 107; bottom: Cam-CAN, *N* = 50) for each modelled cortical region of the Desikan-Killiany atlas. Warmer colors indicate worse temporal stability, and regions left grey did not achieve the indicated level of reliability within the maximum length of data available for the respective participant sample (OMEGA: 120 s; Cam-CAN: 210 s). Note that these are spatial representations of the same data shown in Fig. 2.

To determine whether key demographic and cognitive factors biased the estimation of MEG temporal stability, we computed an *individual stability contribution* score per participant in the Cam-CAN participant sample (Fig. 1B–3), and related these scores to age, sex, handedness, and general cognitive function (as measured by the ACE-R; Mioshi et al., 2006). We used a leave-one-out adaptation of the previously described stability analysis, wherein for each randomization, time-by-ICC models were generated using all but one participant. The median of these ICC values across brain regions was then extracted per time sample and frequency, leading to a single time-by-ICC model per frequency band. The area under the curve (AUC) was calculated for all such models using the *trapz* function in MATLAB. Each missing-participant’s stability model AUC value was subtracted from a full-sample model AUC value generated within the same set of epoch permutations. This resulted in a single ΔAUC score for each Cam-CAN participant per frequency, representing the change in the temporal stability of the model when they were excluded, relative to when using the full sample. The higher the ΔAUC score, the more stable the model when including said participant. These scores were individually regressed against demographic and cognitive data using the *lm* function in *R* (Team R.C., 2017). Bayesian testing of these models was performed using the *BayesFactor* package (Morey et al., 2015).

### 2.7. Code and data availability

Data used in the preparation of this work were obtained from the Cam-CAN repository (available at http://www.mrc-cbu.cam.ac.uk/datasets/camcan/; Shafto et al., 2014; Taylor et al., 2017) and the OMEGA repository (available at https://www.mcgill.ca/bic/resources/omega; Niso et al., 2016). Code for MEG preprocessing and the stability analysis is available at https://github.com/aiwiesman/rsMEG_StabilityAnalysis.

## 3. Results

### 3.1. Temporal stability of intra-session MEG across time and space

The minimal recording durations required to derive robust, stable estimates of MEG spectral features using LCMV beamforming are illustrated for OMEGA (*n* = 107) and Cam-CAN (*n* = 50) in Figs. 2 and 3 and summarized in Tables 1 and 2. Despite substantial differences in sensing technology, recording environment, and participant demographics, the results were similar between both samples across all six frequencies (i.e., median 95% confidence intervals did not diverge at any data length or frequency; Fig. S2). Overall, most spectral features were robustly estimated when derived from 30 to 120 s of data. In the alpha (8–12 Hz) and beta (15–29 Hz) bands, activity in every region showed “excellent” stability (ICC > 0.90) when derived from data durations of 2 min or less (i.e., within the range of the OMEGA data set), while activity in the delta (2–4 Hz), theta (5–7 Hz) and low-gamma (30–59 Hz) bands reached excellent stability in every region with less than 3.5 min of data (i.e., within the range of the Cam-CAN data set). High-gamma (60–90 Hz) neural activity reached “good” stability in every region but one (left pars opercularis) with data durations of 3.5 min or less.

Stability of the data source imaged with dSPM was qualitatively similar to, or perhaps more stable than, those obtained with the LCMV beamformer (Figs. S3 and S4), and the median 95% confidence intervals for the two methods did not diverge across tested data lengths and frequencies (Fig. S5). The inclusion of empty-room data for estimation of noise covariance for dSPM had no discernable effect on stability across frequencies (Fig. S6). We verified that these results were unaffected by possible variability in the efficacy of pre-processing steps for raw recordings of different lengths. We found that stability estimates from the Cam-CAN data were unaffected by the length of raw recordings used for pre-processing and source imaging, as data lengths from 60 s to 150 s (tested in increments of 30 s) indicated no meaningful difference in stability from the full length (480 s) version in all frequencies except high-gamma (Fig. S7). Differences in stability across canonical frequency bands were also not due to differences in bandwidth: the tests conducted with the OMEGA sample across equivalent bandwidths of 10 Hz produced similar stability results (Fig. S8).

### 3.2. Stability of aperiodic and periodic spectral components

**Fig. 4.**
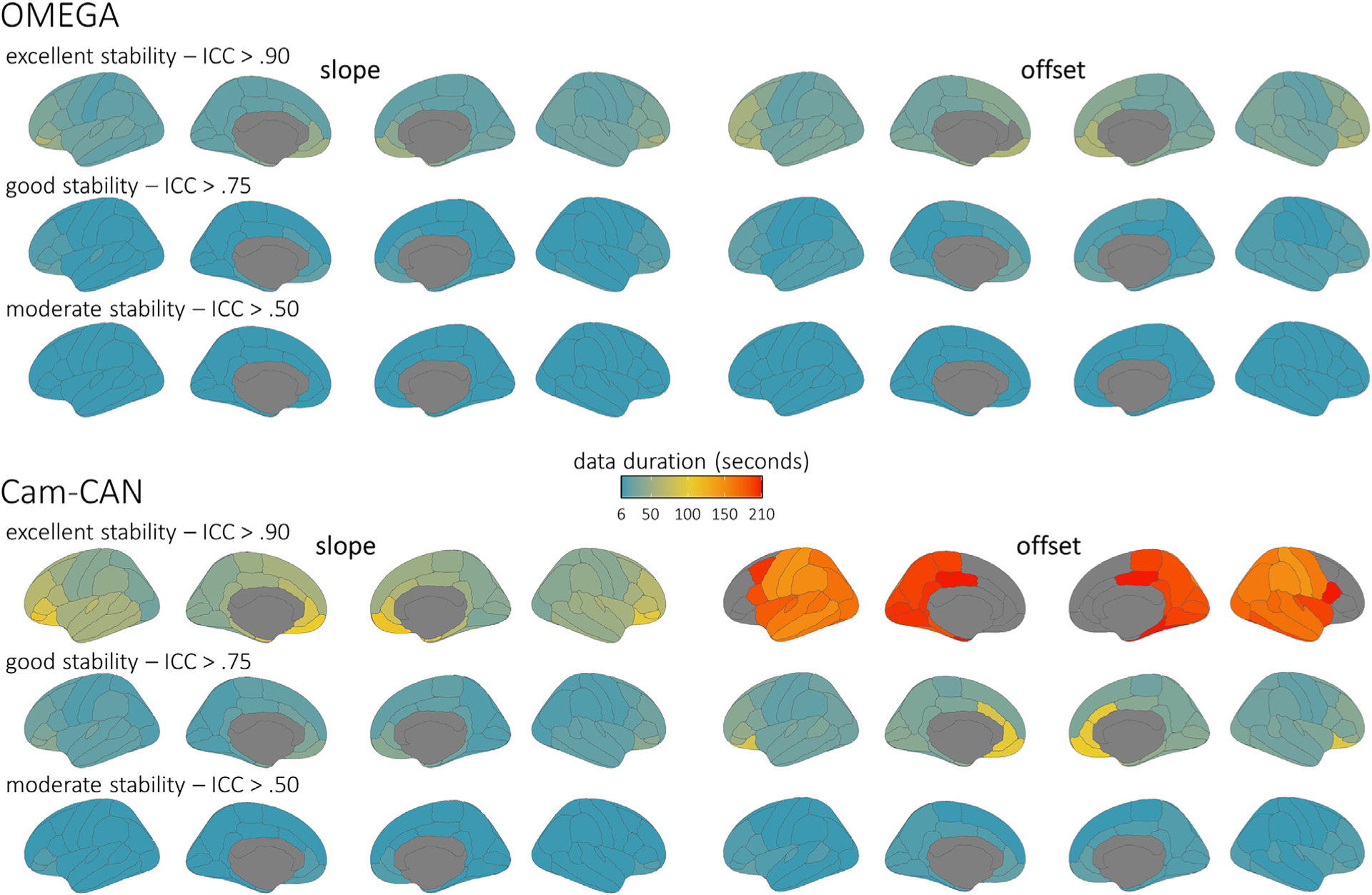
Brain maps of intra-session stability of parameterized aperiodic features. Parcellated surface maps per each aperiodic feature (left: slope; right: offset) indicate the length of data (colour bar; in seconds) required to achieve accepted thresholds for moderate (ICC > 0.50; bottom rows), good (ICC > 0.75; middle rows), and excellent (ICC > 0.90; top rows) reliability in each participant sample (top: OMEGA, *N* = 107; bottom: Cam-CAN, *N* = 50) for each modelled cortical region of the Desikan-Killiany atlas. Warmer colors indicate worse temporal stability, and regions left grey did not achieve the indicated level of reliability within the maximum length of data available for the respective participant sample (OMEGA: 120 s; Cam-CAN: 210 s).

**Fig. 5.**
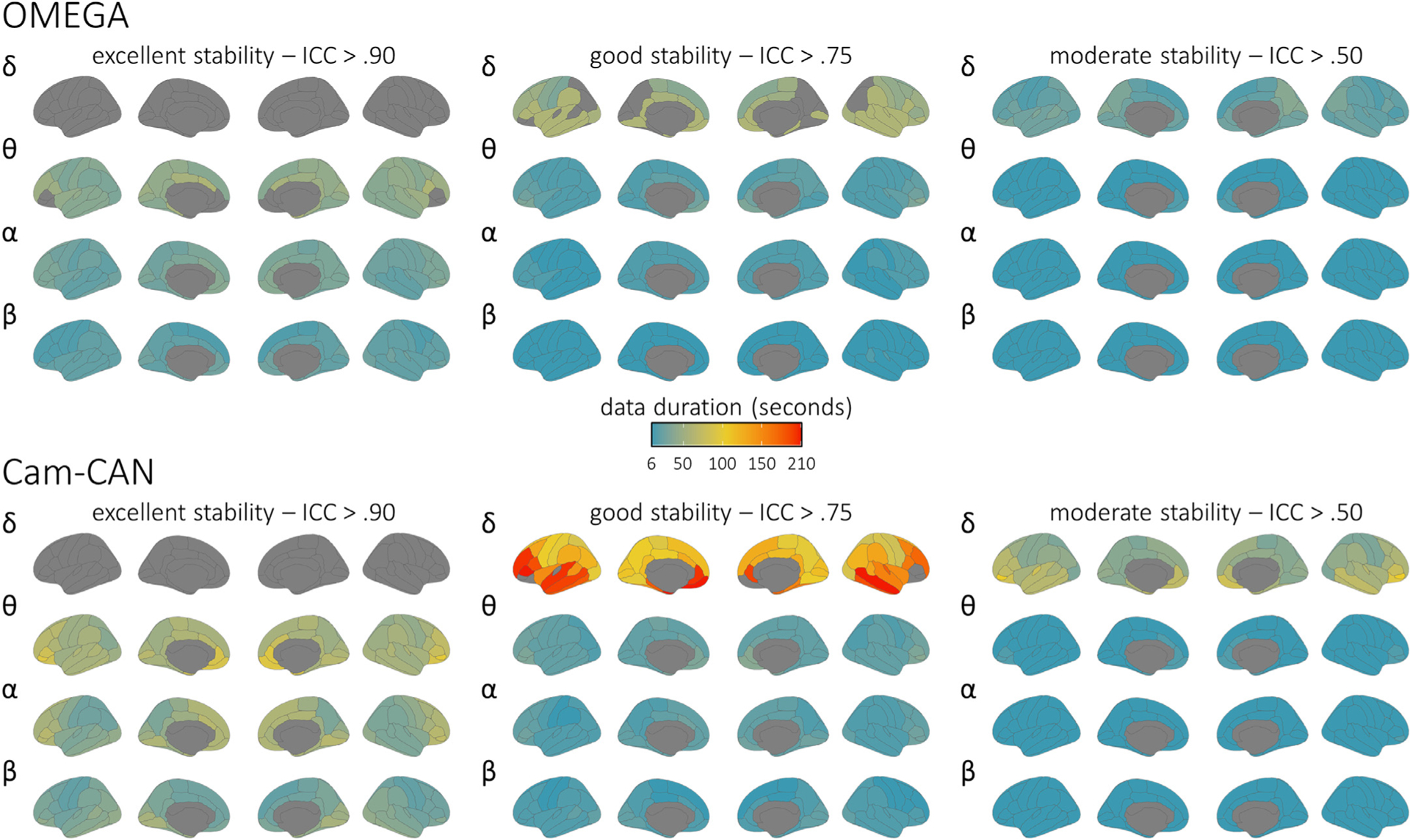
Brain maps of intra-session stability of parameterized periodic features. Parcellated surface maps per each spectral frequency (denoted by Greek letters to the top-left of each set) indicate the length of data (colour bar; in seconds) required to achieve accepted thresholds for moderate (ICC > 0.50; right), good (ICC > 0.75; middle), and excellent (ICC > 0.90; left) reliability in each participant sample (top: OMEGA, *N* = 107; bottom: Cam-CAN, *N* = 50) for each modelled cortical region of the Desikan-Killiany atlas. Warmer colors indicate worse temporal stability, and regions left grey did not achieve the indicated level of reliability within the maximum length of data available for the respective participant sample (OMEGA: 120 s; Cam-CAN: 210 s).

In both the OMEGA and Cam-CAN samples, the estimation of the aperiodic slope achieved excellent stability when derived from less than 2 min of data. However, the estimation of the aperiodic offset was noticeably less robust in Cam-CAN participants, particularly in frontal cortices (Fig. 4). Parameterized periodic neural activity in the theta, alpha, and beta bands reached excellent stability in every region with less than 2 min of data, apart from bilateral inferior and medial frontal regions (Fig. 5), which required slightly longer data durations. In contrast, the periodic delta components were noticeably less stable, and did not reach excellent stability in any region with 3.5 min of data. Importantly, the instability of the delta periodic component was neither due to the length of the epoch nor of the time window used for PSD derivations: similar results were obtained when using 6-s epochs/3-s time windows or 12-s epochs/6-s time windows (Figs. S9 and S10). The instability of the delta periodic component may instead have been caused by intra-session fluctuations in the goodness-of-fit of the FOOOF model at low frequencies. Controlling for regional variations in FOOOF model fit (i.e., R^2)^ across participants in the OMEGA sample markedly improved the robust estimation of periodic activity in the delta band (Figs. S11 and S12).

### 3.3. Influence of demographics and cognitive factors

**Fig. 6.**
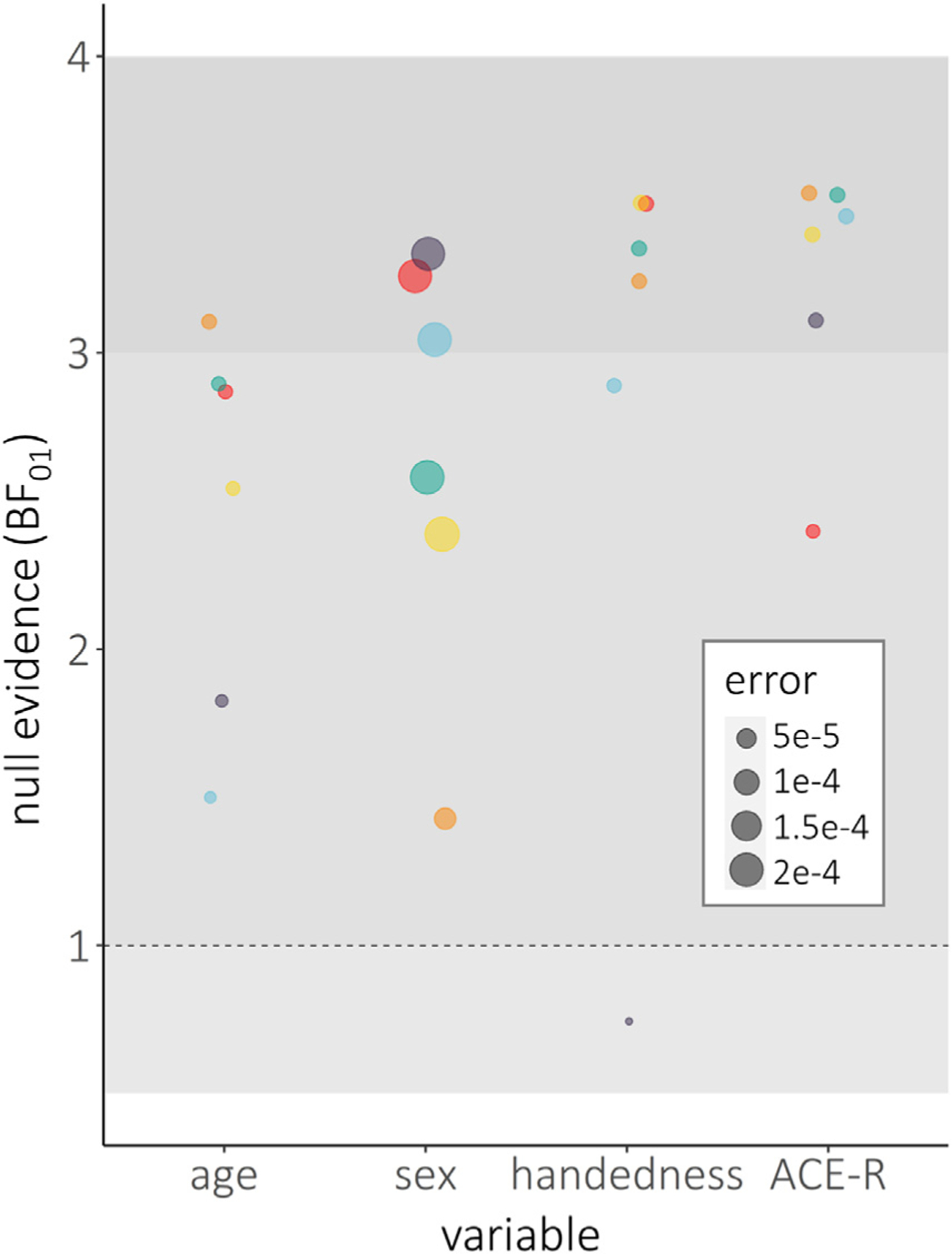
Bayesian model evidence for null effects of participant sample characteristics on the stability of spectral power estimates. Dots indicate Bayesian model evidence (y-axis; in BF01) and associated model errors (denoted by relative dot size) for null effects of common participant sample characteristics (x-axis) on temporal stability for each spectral frequency (denoted by dot colour). Horizontal shaded intervals represent accepted thresholds for weak evidence for H1 (BF01 = 0.3 – 1), weak evidence for H0 (BF01 = 1 – 3), and moderate evidence for H0 (BF01 ≥ 3). Note that these models were only obtained for the Cam-CAN sample, due to its even distribution of age and availability of ACE-R cognitive scores.

We investigated whether common participant sample characteristics impacted the stability of MEG spectral features. Even without correcting for multiple comparisons (6 frequencies × 4 sample characteristics = 24 tests), none of the effects were significant at *p* < .05. Post-hoc Bayesian testing indicated evidence for the null hypothesis in all cases except one (high-gamma & handedness; BF_01_ = 0.75; error% = 7.09 *e*^−6;^ Fig. 6).

## 4. Discussion

Despite a substantial literature examining the relationships between frequency-defined neural activity and various states of cognition and disease, little research exists regarding appropriate data durations to derive robust spectral estimates of human electrophysiology signals. We believe that evidence-based recommendations concerning the minimum data duration required for extracting meaningful spectral features of brain activity are required to guide the design of resting-state studies and inform decisions when growing shared data repositories in the field. This work partially addresses this knowledge gap by quantitatively estimating the intra-session stability over time of source-imaged spectral features of resting-state MEG data.

Overall, we find that spectral estimates of task-free MEG brain activity stabilize remarkably quickly when derived from durations as short as 30 s, and typically 120 s, of data. These results align with recent publications: one reporting stability of EEG graph theory metrics in under 10 s (Fraschini et al., 2016) and another finding that participants can be differentiated from one another based on MEG spectral “fingerprints” obtained with as little as 30 s of resting-state data (da Silva Castanheira et al., 2021). The length of the recording used for initial data preprocessing and source imaging also generally had no effect on stability estimates in truncated recordings as short as 60 s. We noted some exceptions in the high-gamma range. Here, the data duration required to reach stability was longer than for the alpha and beta bands, and the initial recording length used for preprocessing and imaging had a substantive impact on intra-session stability. This indicates that, in cases where shorter MEG recording times are desirable, such as when collecting data from special or patient populations, the length of resting-state recordings could be determined depending on the cortical regions and spectral frequencies of interest. For instance, data durations of at least 2 min of good quality resting-state data would be appropriate for investigations focused on ongoing alpha and beta activity, while studies of ongoing delta/theta/low-gamma activity would require at least 3 min of good resting-state MEG data, and those targeting high-frequency gamma power should aim to record more than 3 min of good-quality data. These recommendations for minimum durations of “good” (i.e., artefact-free, noise-reduced) data can also be extended to minimum recording times for resting-state MEG: we show that preprocessing of shorter raw data lengths has no effect on the stability of spectral derivatives in the delta, theta, alpha, beta, and low-gamma bands. Thus, we conclude that our recommendations for minimum data durations hold, even for recordings as short as 60 s. In contrast, the intra-session stability of high-gamma derivatives varies substantially with raw data length, which provides additional support to the caveat that longer recording times are required to capture the variability of high-frequency activity. Importantly, we also emphasize that spectral instability at higher frequencies should not be conflated to signals being affected by intractable levels of noise. The variability of high-frequency gamma activity is well documented to be associated to various cognitive functions (Başar, 2013; Uhlhaas et al., 2011; Ward, 2003; Wiesman et al., 2020; Wiesman and Wilson, 2019), disease states (Herrmann and Demiralp, 2005; Mably and Colgin, 2018; Uhlhaas and Singer, 2010), and inter-individual differences (da Silva Castanheira et al., 2021; Hirschmann et al., 2020; Muthukumaraswamy et al., 2010; Perry et al., 2013; Shaw et al., 2017). Furthermore, we show that estimates of intra-session resting-state MEG stability in normative populations require samples of at least 50 participants. These recommendations are based on our sub-sampling approach and offer researchers a yardstick when examining stability of spectral power derivatives at the group level.

These stability estimates support and extend existing research on the effects of data duration on neuroimaging signal reliability. Studies of data stability in fMRI have often recommended data durations of more than 10 min (Anderson et al., 2011; Birn et al., 2013; Murphy et al., 2007), which indicates a divergent pattern of intra-session stability for brain hemodynamics. Many of the extant electrophysiology (i.e., MEG and EEG) duration-reliability studies have focused on inter-session effects, which provide information regarding the data durations required for neural features to replicate over long periods of time (i.e., test-retest), as opposed to those required for a stable “snapshot” of brain activity within a single session (i.e., stability). Nevertheless, inter-session M/EEG reliability studies of spectral power derivatives have reported reaching high test-retest reliability with similar data durations as those reported herein (Duan et al., 2021; Gasser et al., 1985; Marquetand et al., 2019; Salinsky et al., 1991; Thatcher, 2010; Van Albada et al., 2007). In contrast, previous intra-session M/EEG reliability studies have exclusively examined connectivity metrics and reported mixed results. Fraschini et al. (2016) indicate high levels of stability for EEG connectivity graph metrics with less than 10 s of data in some cases, while Liuzzi et al. (2017) report significantly higher within-session reliability of MEG estimates derived from 280 versus 120 s of data. While this discrepancy deserves further investigation, reduced intra-session reliability of connectivity estimates is perhaps intuitive, as the stability of these metrics inherently requires robust estimation of neural activity in two regions rather than one, as well as the statistical associations between them.

We also found that the stability of spectral features was similar between both MEG instruments tested (CTF vs. MEGIN/Elekta), sampling rates used in data collection (2400 Hz vs. 1000 Hz), and resting-state paradigms (i.e., eyes-open vs. eyes-closed). We acknowledge the present study was not exhaustive with regards to all possible experimental factors that might influence MEG signal stability, but the MEG instruments and paradigmatic approaches encompass those most employed in the field. This is in agreement with a recent study showing similar longitudinal test-retest reliability of MEG derivatives, regardless of instrument type (Boon et al., 2021). The similarity of results from data collected with different sampling rates also suggests that it is the absolute temporal duration (e.g., in seconds), rather than the number of data samples, that dictates the stability of spectral MEG features, at least when sampling at or above 1000 Hz. Differing approaches to source imaging (i.e., LCMV beamforming versus dSPM) also produced highly similar stability estimates across data durations and frequencies. In fact, the derivatives from data source imaged with dSPM qualitatively appeared more stable than those imaged with LCMV, indicating that our original recommendations for minimum data durations based on the LCMV stability estimates are, if anything, conservative. Additionally, the stability of spectral features was unaffected by common participant characteristics such as age, sex, handedness, and cognitive status. We emphasize that these factors are of research interest but evidence from our results indicate they may not require different durations of signal acquisition in the resting-state of healthy participants.

We also examined the robustness across varying data lengths of estimates of aperiodic and periodic spectral components of neurophysiological resting-state time series. The estimates of periodic components stabilized (ICC > 0.90) when derived from less than two minutes of data in the alpha and beta bands across the brain, and within 3.5 min of data in the theta band. We note that the theta, alpha, and beta bands are the most susceptible to exhibiting separable peaks in the human neurophysiological spectrum (Donoghue et al., 2020). This contrasts with parameterized delta activity estimates, which did not reach excellent temporal stability in any region with less than 3.5 min of data. This lack of robustness was not explained by the short epoch duration and window length used for PSD derivations: using longer epochs (12 s) and window lengths (6 s) produced equivalent results. Rather, we found that the instability of low-frequency spectral estimates was due to variability of FOOOF models across participants. This indicates that the FOOOF algorithm is challenged in accounting for the periodic components in the delta band because it may be too close to the lower edge of the frequency spectrum, that the noise floor of MEG sensors is slightly higher at delta frequencies, and/or that delta rhythmic activity is uncommon in healthy participants and therefore that estimates of periodic activity may be spurious. This latter point aligns with the small number of delta peaks in resting-state data reported in the original FOOOF study (Donoghue et al., 2020). Overall, low-frequency estimates of parameterized periodic spectral components in resting-state MEG are highly variable over time within typical session durations and should be interpreted with caution.

We found that estimates of the aperiodic slope were robust when derived from at least two minutes of OMEGA and Cam-CAN data. In contrast, the stability of the aperiodic offset differed between the OMEGA and Cam-CAN samples. Whereas the estimation of the spectral offset was stable for OMEGA data durations of about two minutes, its estimation from Cam-CAN data required longer durations and offset estimates in several frontal and medial regions did not reach excellent stability. We are unable to empirically determine the origin of this discrepancy in this study, as many experimental parameters (e.g., MEG instrument, resting-state paradigm, and noise-cancellation approach) were different between the OMEGA and Cam-CAN samples. However, we can speculate that the parameters that most likely influence the low-frequency offset are the noise-cancellation method (i.e., third-order synthetic gradiometers versus MaxFilter spatial filter) and the resting-state paradigm (i.e., eyes-open vs. eyes-closed). The estimation of the aperiodic offset is highly dependent on the correct estimation of lower-frequency periodic components, namely from the delta band, in which stability was also challenged in these cortical regions. Future research is needed to explore the sources of this disagreement between the OMEGA and Cam-CAN samples.

It should be noted that some of our recommendations are predicated on the premise of equivalent stability between the OMEGA and Cam-CAN samples, as the OMEGA recordings (~4 min) were substantially shorter in duration than those collected for Cam-CAN (~8 min). This is supported by our finding that the median 95% confidence intervals for the stability of these samples overlapped at every frequency and data duration; however, the high dimensionality of our data precluded us from performing this analysis at every brain region. Thus, it remains possible that specific combinations of region, frequency, and data duration might exhibit higher stability in one sample versus the other. Theta frequency derivatives from the right superior temporal sulcus, for example, appear to reach excellent stability before 120 s in the Cam-CAN sample, but did not reach an equivalent level of stability in the 120 s of the OMEGA data. In cases where planned research will focus on these regions of non-overlap in our findings, we conservatively recommend the use of longer recording durations to ensure intra-session data stability. We also found that the estimation of high-gamma spectral power was not stable over the recording durations available from OMEGA and Cam-CAN. We can extrapolate from our data that future studies will require MEG resting-state recordings of at least 10 min per participant to derive stable estimates of high-gamma power (Fig. S1). This pattern of high intra-session gamma variability and low intra-session alpha and beta variability is similar to a recent study of long term (i.e., 3 year) inter-session variability of MEG spectral power (Lew et al., 2021). This suggests that alpha and beta spectral power represent a reliable estimate of inter-participant neural variability, both within a single-session “snapshot” of participant neural activity and across sessions separated even by multiple years. We are also aware that our findings may not generalize to all populations. These recommendations are based on the amount of artefact-free/useable signal available in each recording, which is often unpredictable and may be relatively lower in e.g., younger children or specific patient populations. The variability of MEG data may also be different in certain participant groups for reasons related to actual differences in neurophysiological activity due to age or development of pathology. It is therefore advisable to record additional data to account for such eventuality. Finally, we did not test for biases in our data due to variations in the signal-to-noise (SNR) of our source estimates, which might be expected to artificially inflate signal stability in regions with worse SNR (i.e., due to the increased smoothness of data in these regions). While we cannot rule out this possibility, we think it is unlikely that our results are biased by SNR, as indicated by the spatial distributions of temporal stability across the brain. For example, if SNR would bias spatial variability, one would expect regions/frequencies with higher SNR (e.g., alpha activity in parieto-occipital cortices, theta activity in frontal regions, beta activity in somato-motor regions) to exhibit lower stability. Across the tested sensor technologies (i.e., MEG instruments), recording paradigms, and source imaging methods, no such bias appears to exist in our data.

Considering these limitations, we look at our present findings as a normative benchmark for neuroscience studies of resting-state brain activity. We hope that our recommendations will prove particularly useful to researchers when designing their MEG resting-state studies and extracting spectral features. Amidst concerns about the reproducibility and replicability of findings across scientific fields (Loken and Gelman, 2017), it may be reassuring that common estimators of spectral properties of brain signals are robust when derived from recording durations typically used in a large majority of existing research and open datasets.

## Supplementary Material

1

## Figures and Tables

**Table 1:** Values are data durations (in seconds) required to reach specified levels of excellent, good, and moderate stability. Median, minimum, and maximum values were computed across brain regions, and the most and least stable regions were defined as the regions that exhibited the highest and lowest overall intraclass correlations, respectively, across all possible data durations. DNS: did not stabilize to specified ICC level within available data durations. ICC: intraclass correlation coefficient. OMEGA: Open MEG Archives participant subsample.

OMEGA	excellent – ICC > 0.90			good – ICC > 0.75			moderate – ICC > 0.50			most stable region	least stable region
	median	min	max	median	min	max	median	min	max		
power spectral density											
delta (2–4 Hz)	72	42	*DNS*	24	12	60	6	6	18	postcentral L	frontalpole L
theta (5–7 Hz)	96	66	*DNS*	30	24	48	12	6	18	precentral L	parahippocampal R
alpha (8–12 Hz)	72	54	114	24	18	36	12	6	12	parsorbitalis R	cuneus L
beta (15–29 Hz)	33	18	60	12	6	18	6	6	6	temporalpole L	supramarginal R
low-gamma (30–59 Hz)	72	12	*DNS*	24	6	*DNS*	6	6	78	superiorparietal L	parsopercularis R
high-gamma (60–90 Hz)	*DNS*	42	*DNS*	111	12	*DNS*	36	6	*DNS*	superiorparietal L	parstriangularis R
parameterized periodic											
delta (2–4 Hz)	*DNS*	*DNS*	*DNS*	114	48	*DNS*	39	18	72	postcentral L	frontalpole L
theta (5–7 Hz)	69	36	*DNS*	24	12	54	6	6	18	lateraloccipital L	frontalpole L
alpha (8–12 Hz)	42	30	72	12	12	24	6	6	12	superiortemporal L	frontalpole L
beta (15–29 Hz)	30	18	54	12	6	18	6	6	6	precentral L	frontalpole R
parameterized aperiodic											
slope	36	24	*DNS*	12	6	42	6	6	18	postcentral R	frontalpole R
offset	45	30	120	18	12	42	6	6	12	postcentral R	frontalpole L

**Table 2:** Values are data durations (in seconds) required to reach specified levels of excellent, good, and moderate stability. Median, minimum, and maximum values were computed across brain regions, and the most and least stable regions were defined as the regions that exhibited the highest and lowest overall intraclass correlations, respectively, across all possible data durations. DNS: did not stabilize to specified ICC level within available data durations. ICC: intraclass correlation coefficient. Cam-CAN: Cambridge Centre for Ageing and Neuroscience participant subsample.

Cam-CAN	excellent – ICC > 0.90			good – ICC > 0.75			moderate – ICC > 0.50			most stable region	least stable region
	median	min	max	median	min	max	median	min	max		
power spectral density											
delta (2–4 Hz)	96	66	*DNS*	30	24	66	12	6	24	lateraloccipital L	frontalpole R
theta (5–7 Hz)	66	48	126	24	18	36	6	6	12	rostralmiddlefrontal L	cuneus L
alpha (8–12 Hz)	72	48	102	24	18	30	12	6	12	frontalpole R	pericalcarine R
beta (15–29 Hz)	30	18	54	12	6	18	6	6	6	frontalpole R	superiorparietal L
low-gamma (30–59 Hz)	36	6	150	12	6	48	6	6	12	cuneus L	parsopercularis L
high-gamma (60–90 Hz)	141	6	*DNS*	42	6	*DNS*	12	6	90	superiorparietal R	parsopercularis L
parameterized periodic											
delta (2–4 Hz)	*DNS*	*DNS*	*DNS*	153	84	*DNS*	54	30	132	superiorparietal R	frontalpole L
theta (5–7 Hz)	60	36	132	18	12	48	6	6	18	inferiorparietal L	frontalpole R
alpha (8–12 Hz)	48	24	126	18	6	42	6	6	18	supramarginal L	frontalpole R
beta (15–29 Hz)	30	18	60	12	6	18	6	6	6	precentral R	frontalpole L
parameterized aperiodic											
slope	48	24	150	18	12	48	6	6	18	lateraloccipital L	frontalpole R
offset	198	150	*DNS*	30	18	108	12	6	18	postcentral R	medialorbitofrontal R

## Data Availability

Data used in the preparation of this work were obtained from the Cam-CAN repository (available at http://www.mrc-cbu.cam.ac.uk/datasets/camcan/; Shafto et al., 2014; Taylor et al., 2017) and the OMEGA repository (available at https://www.mcgill.ca/bic/resources/omega; Niso et al., 2016). Code for MEG preprocessing and the stability analysis is available at https://github.com/aiwiesman/rsMEG_StabilityAnalysis.
