# Supplementary material for: Stability of spectral estimates in resting-state magnetoencephalography: Recommendations for minimal data duration with neuroanatomical specificity
Source: Neuroimage. Author manuscript; Available in PMC 2022 Feb 17. (PMC8852336; doi:10.1016/j.neuroimage.2021.118823)
Supplement: 1 [file NIHMS1774999-supplement-1.pdf]

Supplementary Figures

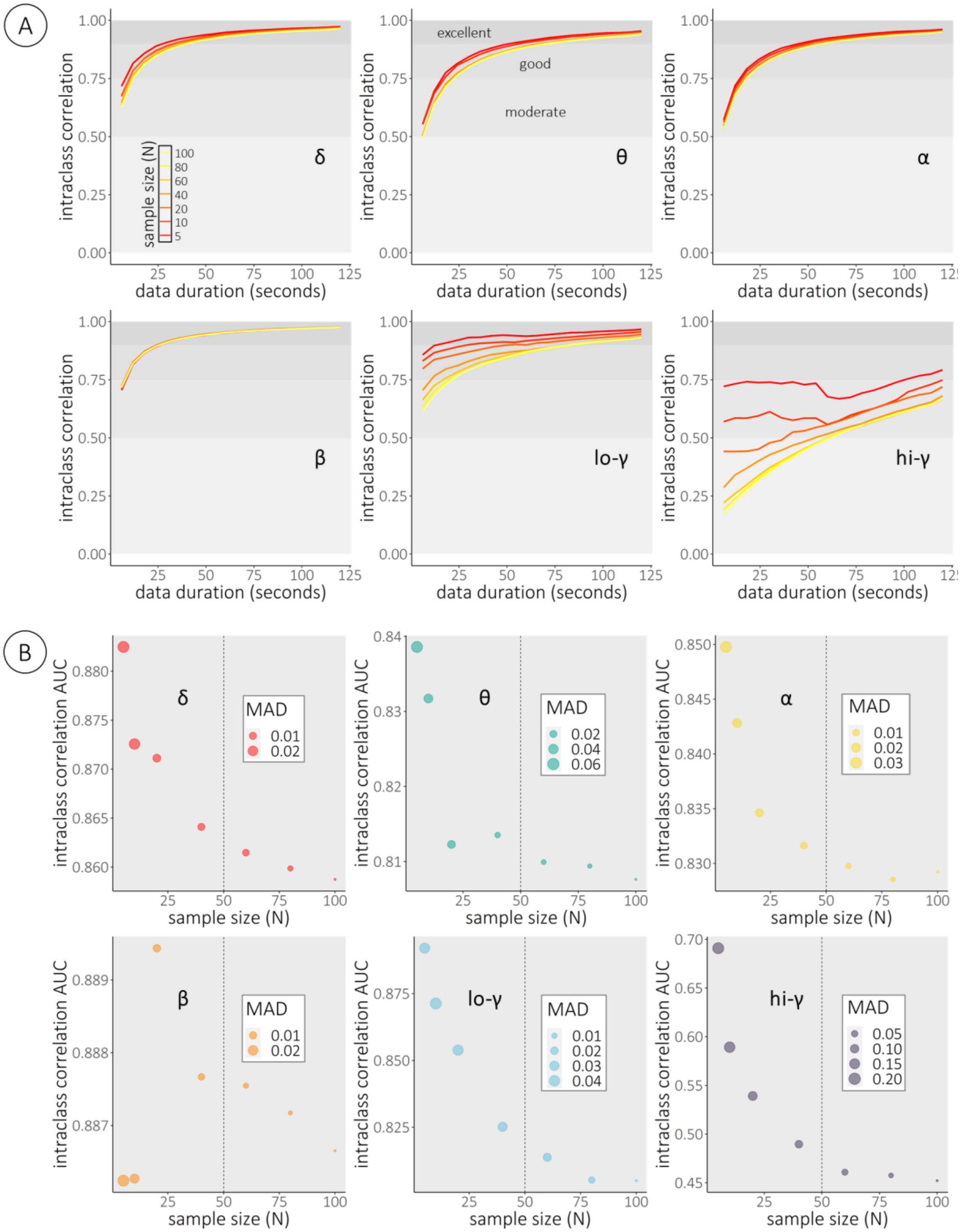

Figure S1. Intra-session temporal stability time series per sample size and spectral frequency. (A) Intraclass correlation coefficients (y-axes) are medians over 1,000 permutations of epoch order, and represent the stability of each neural power estimate, averaged over all cortical regions, as a function of participant sample size (denoted by line color; median over 100 permutations of participants), length of data (x-axes; in seconds), and spectral frequency (separate plots; denoted by Greek letters in the top left of

each). Horizontal shaded intervals in each plot represent accepted thresholds for moderate (ICC > .50), good (ICC > .75), and excellent (ICC > .90) reliability. (B) Intraclass correlation area-under-the-curve (AUC) values are integrals of the curves in (A), and dot sizes represent integrals for similar median absolute deviation (MAD) curves. The y-axes in (B) vary across each frequency sub-plot to accentuate effects of sample size. Note that this modeling was only performed in the OMEGA sample, to quasi-empirically estimate the number of participants that would subsequently be needed from the Cam-CAN dataset.

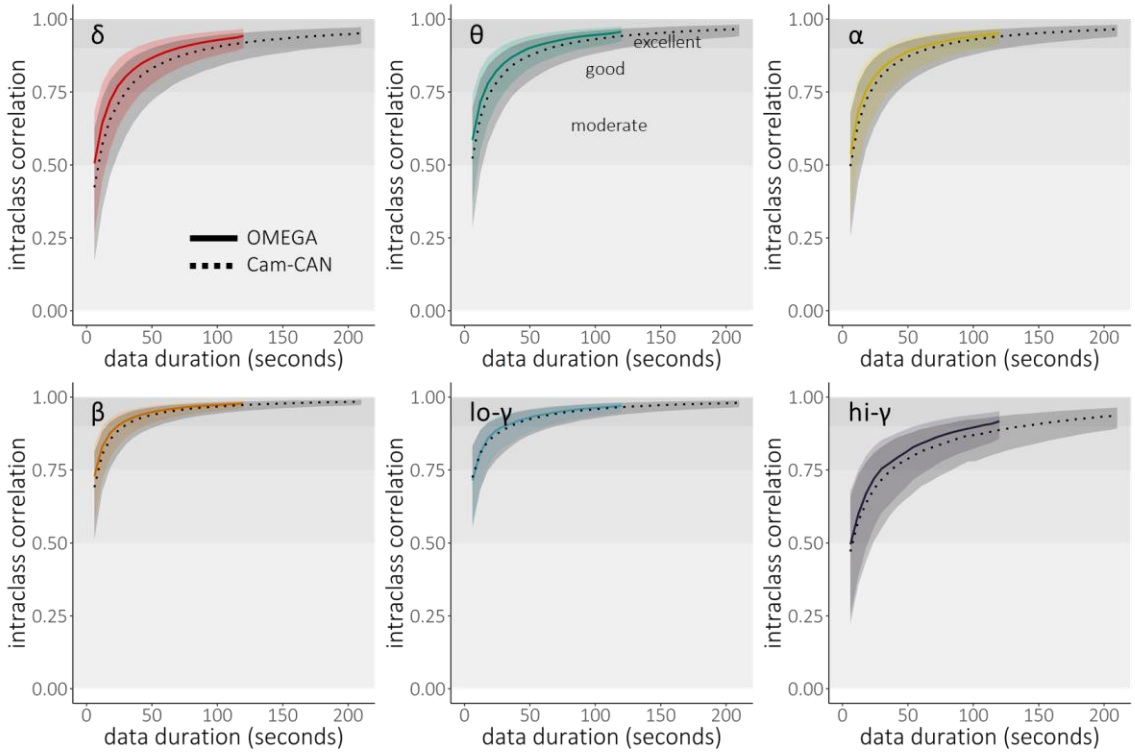

Figure S2. Comparisons of band-limited power stability between the OMEGA and Cam-CAN samples. Median 95% confidence intervals for the intraclass correlations from the analyses in Figure 2 were computed in the OMEGA and Cam-CAN samples to facilitate their direct comparison at each frequency. Line plots indicate the median intraclass correlation coefficients across cortical regions for the OMEGA (colored lines) and Cam-CAN (dotted lines) samples. Shaded intervals represent median 95% confidence intervals across regions for each sample.

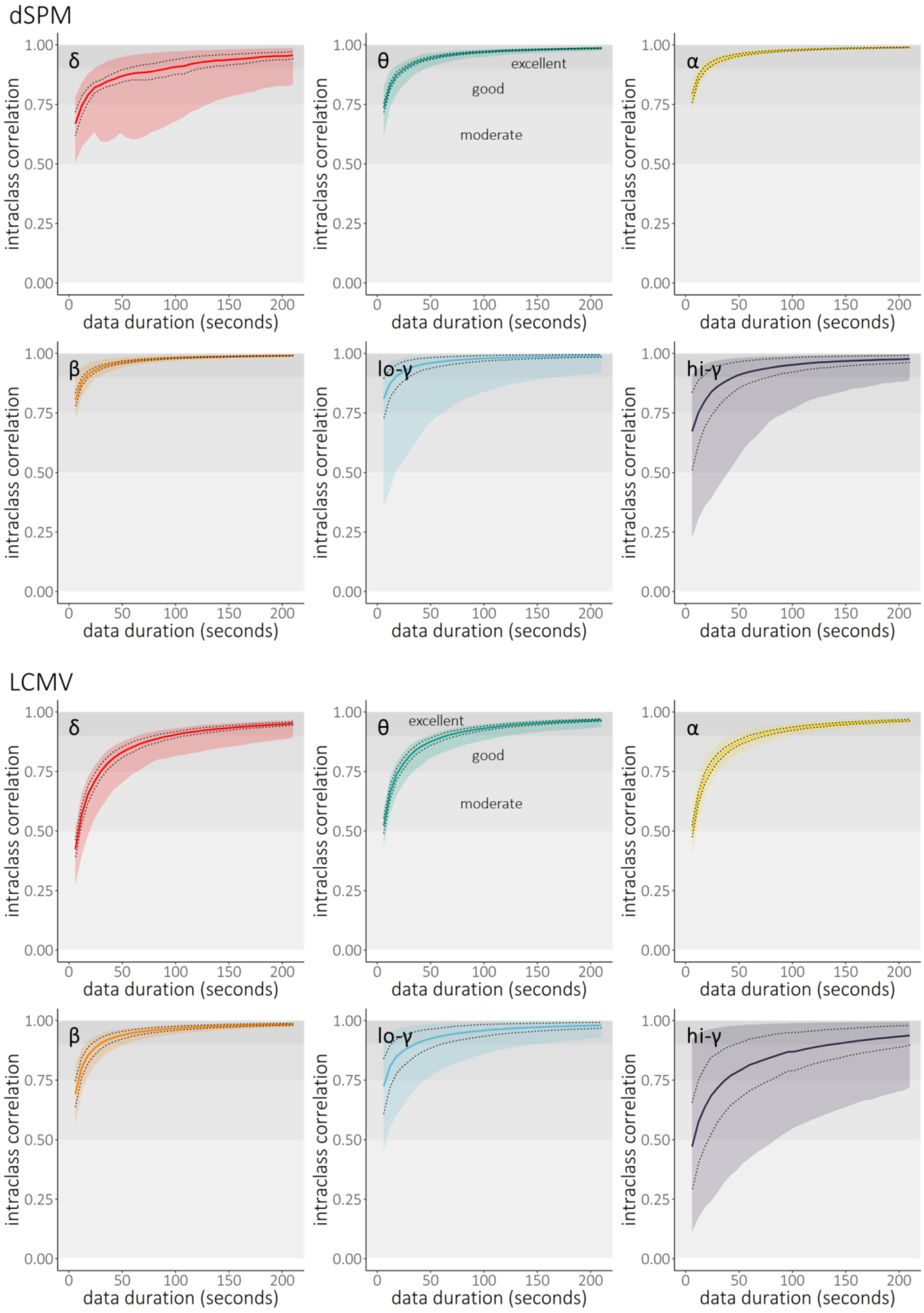

Figure S3. Temporal stability of band-limited power estimates generated with different source-imaging approaches. The analyses from Figure 2 were recomputed in the Cam-CAN sample on data source-imaged with another widely-used approach: dynamic

statistical parametric mapping (dSPM; top). The Cam-CAN results from Figure 2 (imaged with the linearly constrained minimum variance beamformer; LCMV) are plotted below, to facilitate comparison. Colored lines represent the median across regions, dotted lines indicate ± one median absolute deviation across regions, and colored shaded intervals represent the range of stability values across all modeled cortical regions of the Desikan-Killiany atlas.

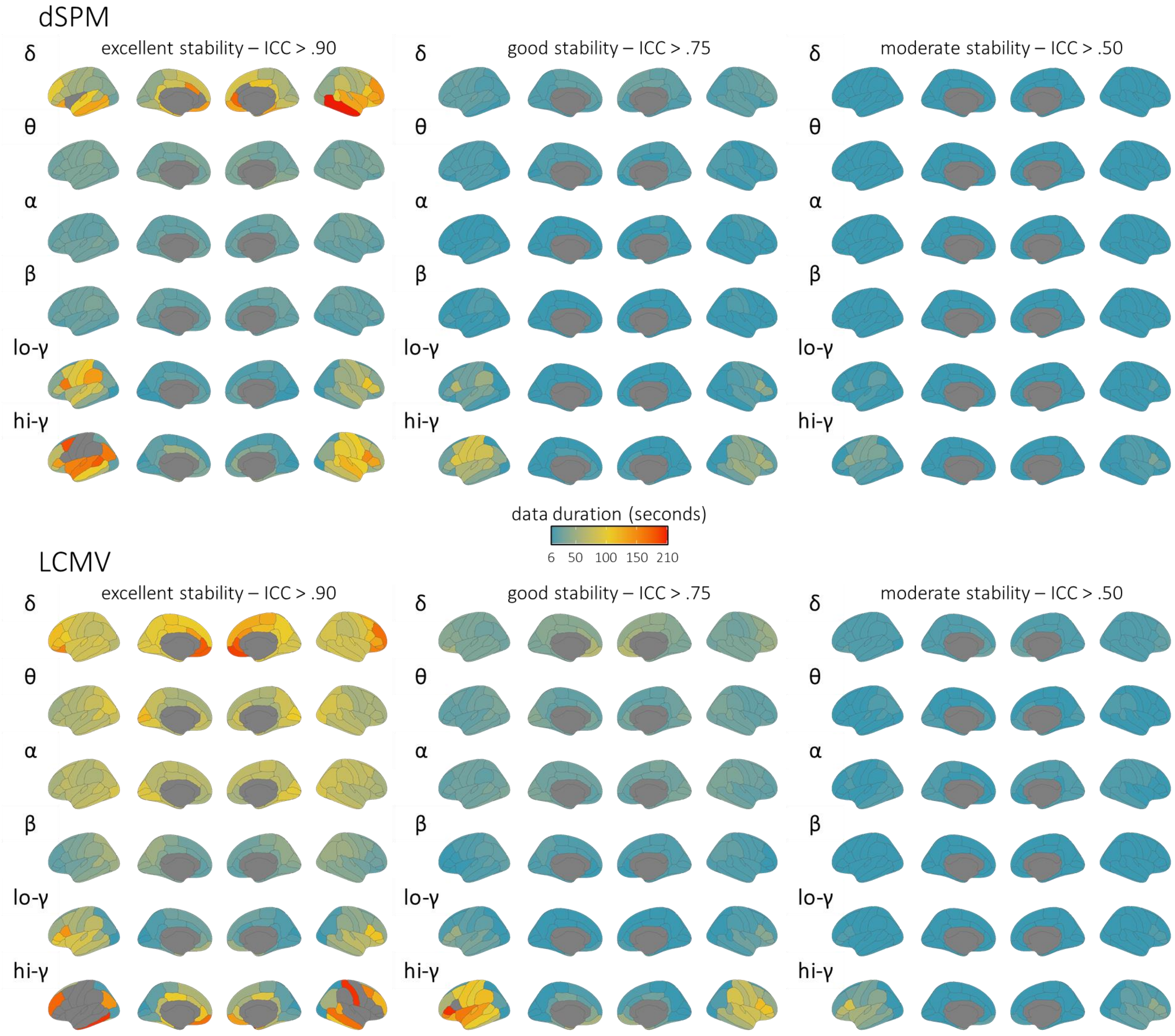

Figure S4. Brain maps of band-limited power temporal stability generated with different source-imaging approaches. The analyses from Figure 3 were recomputed in the Cam-CAN sample on data source-imaged with dynamic statistical parametric mapping (dSPM; top). Parcellated surface maps below are equivalent to the Cam-CAN surface maps in Figure 3 (imaged with the linearly constrained minimum variance beamformer; LCMV), to facilitate comparison.

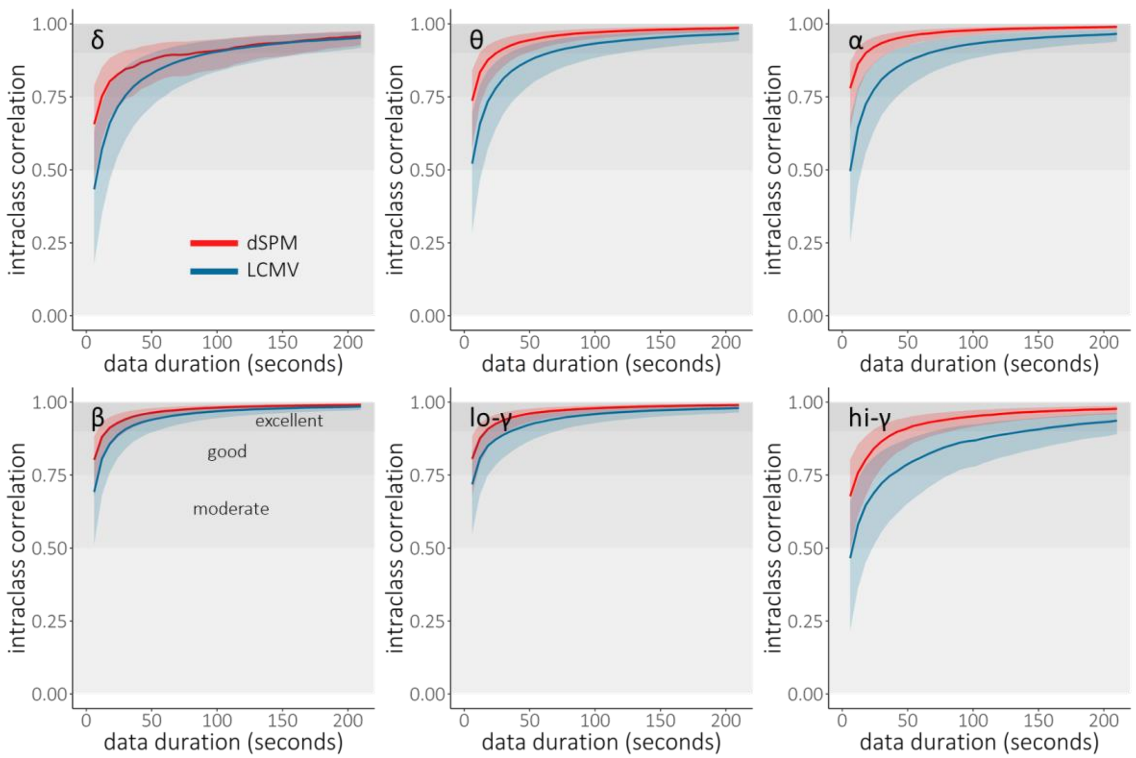

Figure S5. Comparisons of band-limited power stability generated with different source-imaging approaches. The analyses from Figure 2 were recomputed in the Cam-CAN sample on data source-imaged with dynamic statistical parametric mapping (dSPM; red) and linearly constrained minimum variance beamforming (LCMV; blue). Line plots indicate the median intraclass correlation coefficients across cortical regions and shaded intervals represent median 95% confidence intervals across regions.

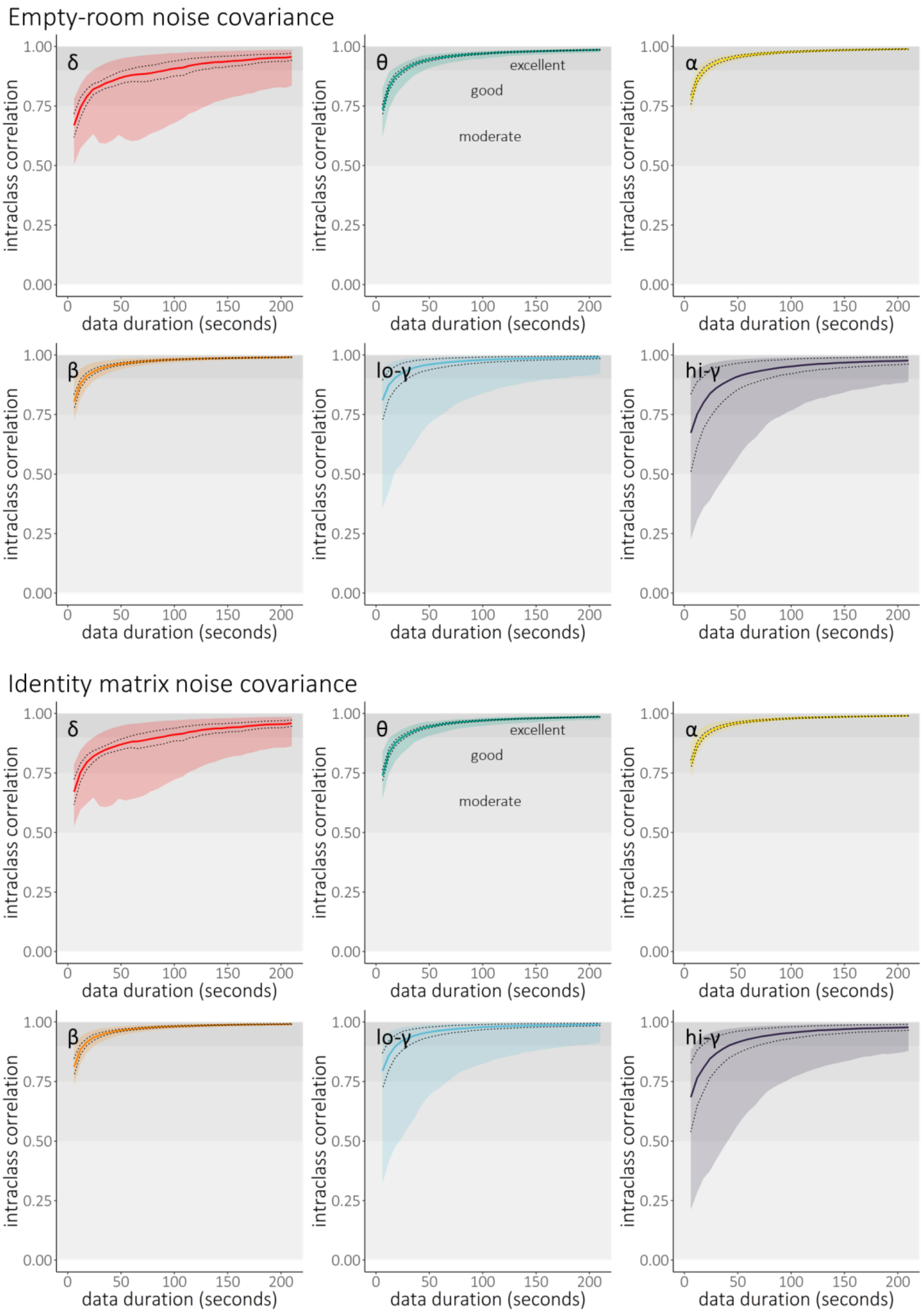

Figure S6. Temporal stability of band-limited power estimates source-imaged with different approximations of the noise covariance. The analyses from Figure S3 were recomputed on data source imaged with dynamic statistical parametric mapping

(dSPM) using different approximations of the noise covariance, including one generated empirically from the statistics of a short (> 120 seconds) empty-room recording (top) and one consisting of an identity matrix (bottom). Colored lines represent the median across regions, dotted lines indicate ± one median absolute deviation across regions, and colored shaded intervals represent the range of stability values across all modeled cortical regions of the Desikan-Killiany atlas.

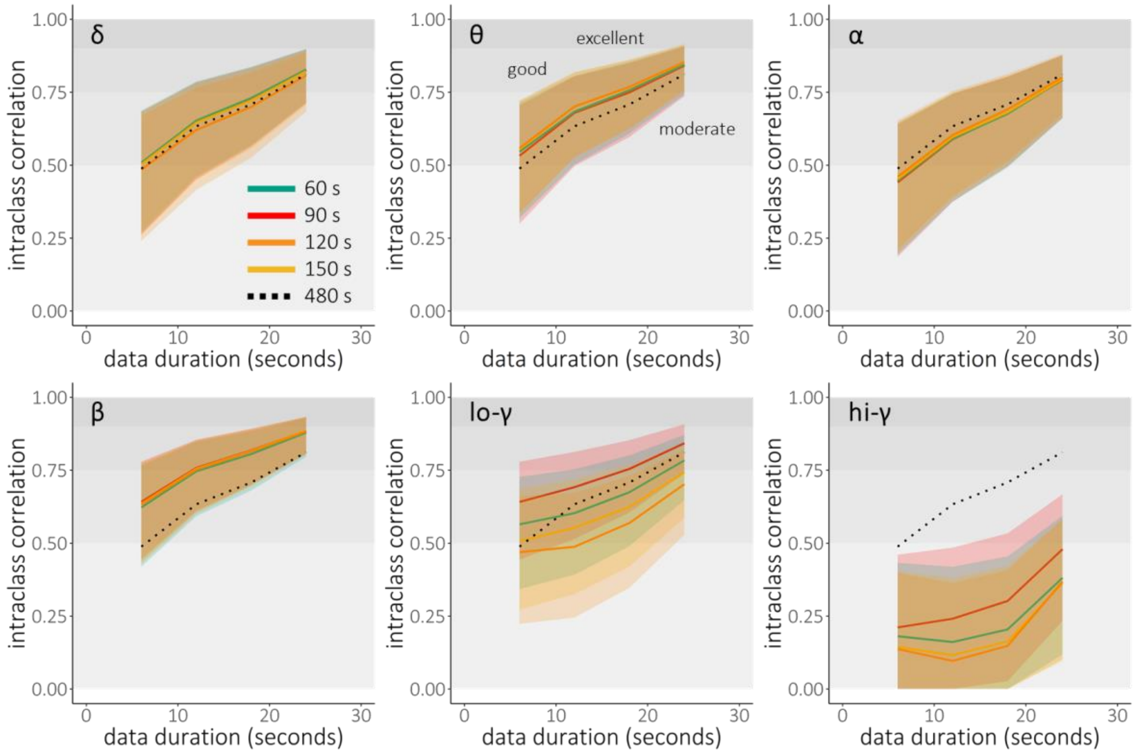

Figure S7. Impact of initial recording length on the intra-session temporal stability of band-limited power. To examine the potential interaction of the initial recording length and major preprocessing steps on band-limited intra-session temporal stability, the analyses from Figure 2 were recomputed in the Cam-CAN sample across data sets that were truncated to various lengths before preprocessing. To facilitate direct comparison, the same 8 epochs were used for each variation, limited by the shortest version (60 seconds). Line plots indicate the median intraclass correlation coefficients (ICC) across cortical regions for the truncated data lengths (colored lines), and shaded intervals represent median 95% confidence intervals across regions for each version. The overlayed dotted lines represent the comparable median ICC values generated from the full-length (480+ seconds) Cam-CAN data shown in Figure 2.

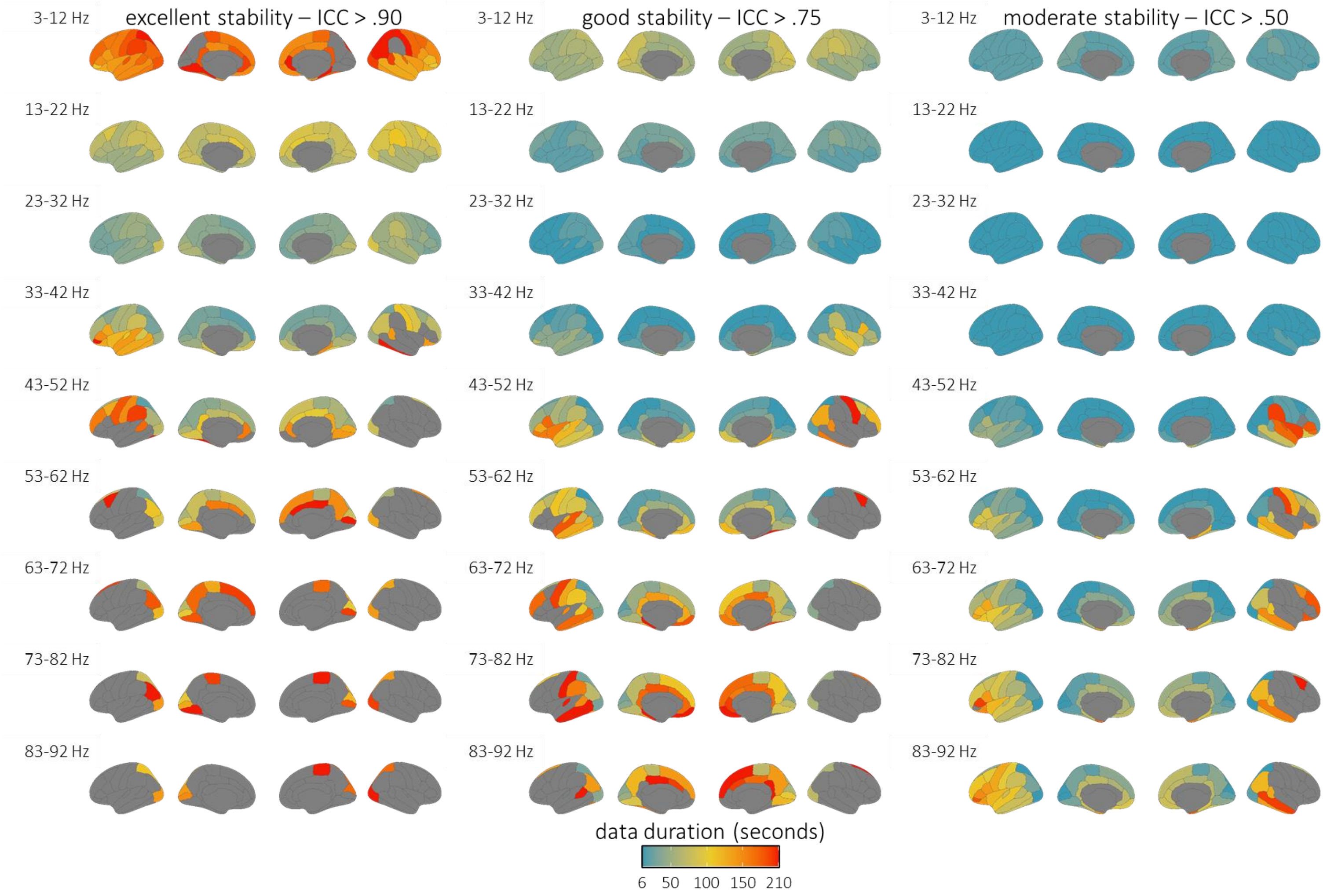

Figure S8. Regional intra-session temporal stability per participant sample and 10 Hz spectral band. Parcellated surface maps are equivalent to the OMEGA surface maps in Figure 3, but computed on data averaged over equivalently-sized 10 Hz spectral definitions (denoted by the text to the top-left of each set), rather than canonical frequency bands.

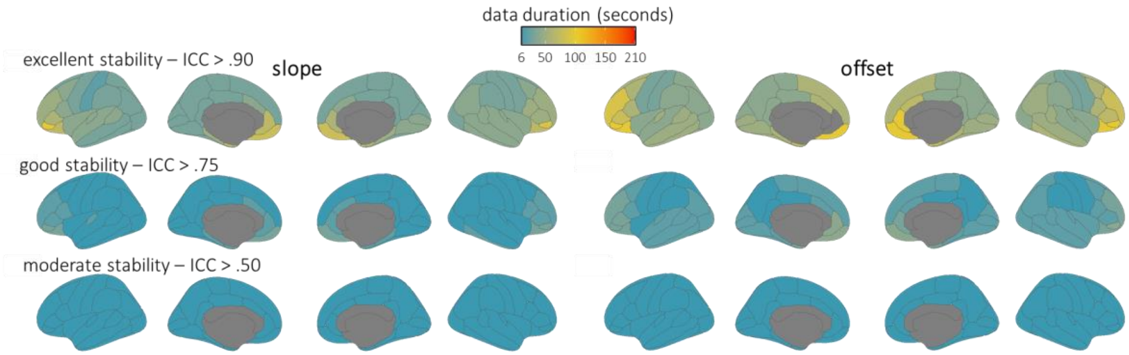

Figure S9. Regional intra-session temporal stability of parameterized aperiodic features using 12 seconds epochs. Parcellated surface maps are equivalent to the OMEGA surface maps in Figure 4, but computed using longer epochs and PSD windows (original: epoch = 6 seconds, PSD window = 3 seconds; new: epoch = 12 seconds; PSD window = 6 seconds).

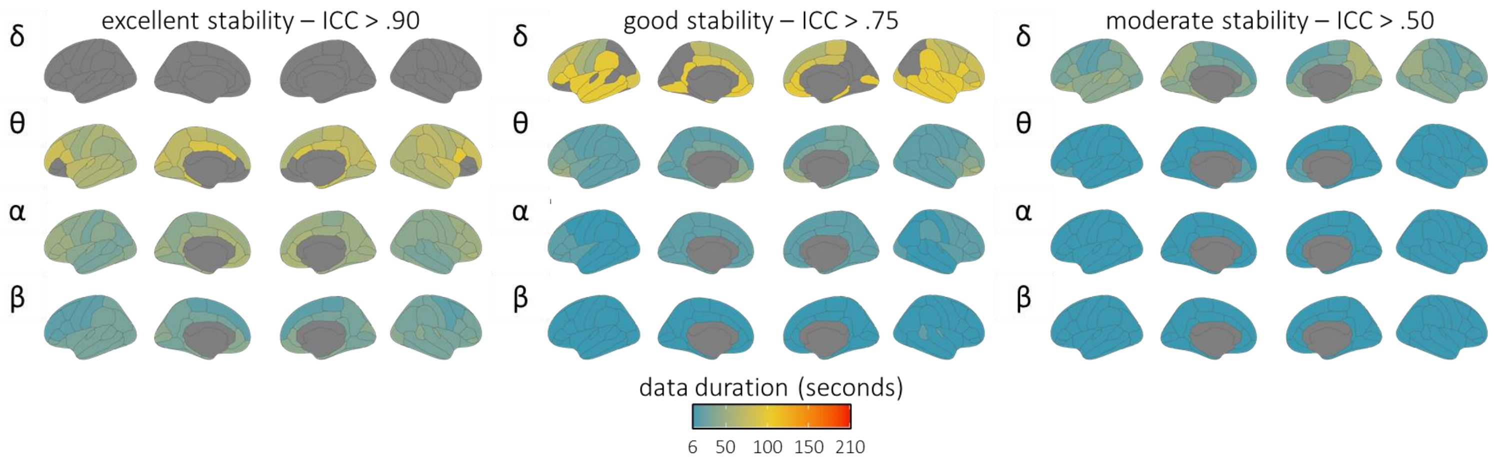

Figure S10. Regional intra-session temporal stability of parameterized periodic features using 12 seconds epochs. Parcellated surface maps are equivalent to the OMEGA surface maps in Figure 5, but computed using longer epochs and PSD windows (original: epoch = 6 seconds, PSD window = 3 seconds; new: epoch = 12 seconds; PSD window = 6 seconds).

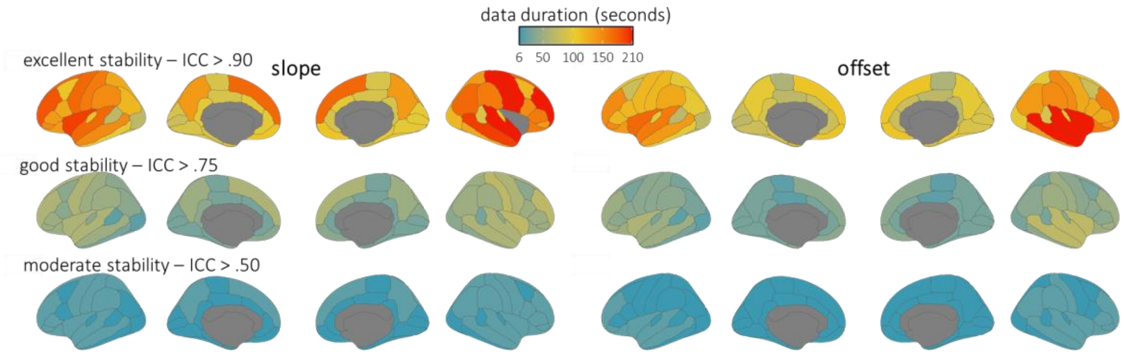

Figure S11. Regional intra-session temporal stability of parameterized aperiodic features covarying FOOOF model fit. Parcellated surface maps are equivalent to the OMEGA surface maps in Figure 4, but computed on the residuals from regressions of parameter estimates on FOOOF model fit (R2) across participants, per each cortical region.

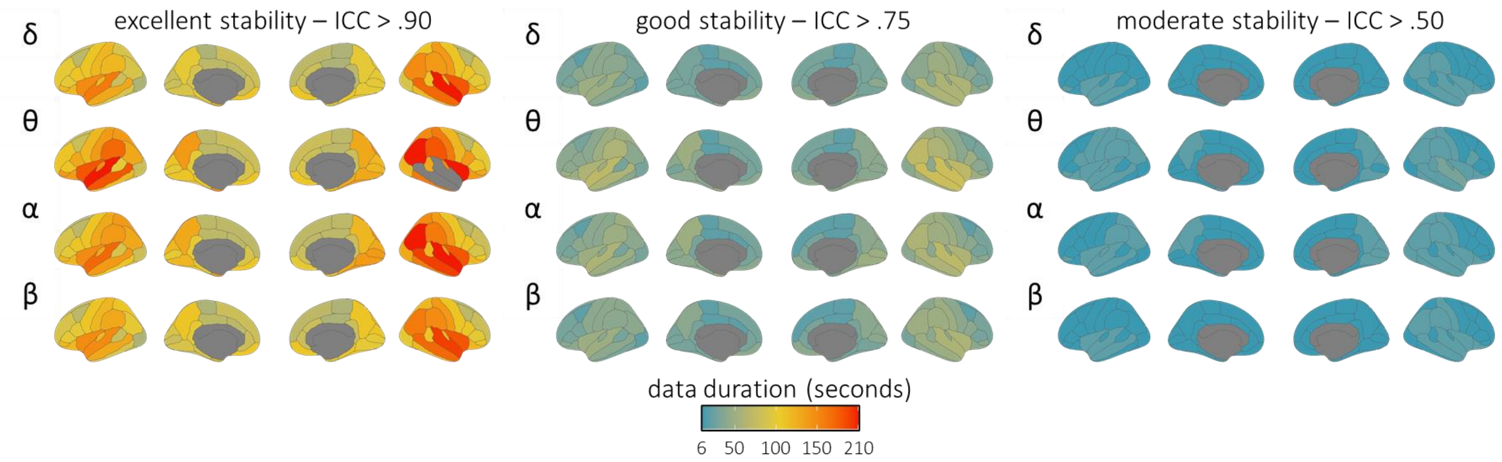

Figure S12. Regional intra-session temporal stability of parameterized periodic features covarying FOOOF model fit. Parcellated surface maps are equivalent to the OMEGA surface maps in Figure 5, but computed on the residuals from regressions of parameter estimates on FOOOF model fit (R2) across participants, per each cortical region.
